# Unusual Penetrating Orbital and Globe Injury With a Veterinary Needle

**DOI:** 10.7759/cureus.48076

**Published:** 2023-10-31

**Authors:** Abeer Alawi, Fawzia AlHaimi

**Affiliations:** 1 Pediatric Ophthalmology and Strabismus Division, King Khaled Eye Specialist Hospital, Riyadh, SAU

**Keywords:** eye emergency, needle type, orbital complications, retina surgery, open globe injury

## Abstract

We present a rare case of a penetrating injury to the globe and orbit by a veterinary needle. The patient underwent globe exploration and retinal detachment repair under general anesthesia after receiving medical treatment for orbital cellulitis. The case was managed by a multidisciplinary team consisting of a pediatric ophthalmologist, an oculoplastic surgeon, and a retina surgeon. The orbital cellulitis resolved gradually, after which the site of globe penetration was found to be self-sealed, and the retinal detachment was repaired. B-scan ultrasonography and magnetic resonance imaging (MRI) were performed to assess the extent of the injury and evaluate the integrity of the globe. Exploration of presumed Self-sealing globe wounds by a needle should be deferred until the treatment of life-threatening complications like orbital cellulitis and optic nerve infiltration is complete. Prompt judicious care was necessary in this case. It is crucial to implement preventive measures to address the risks involved in children manipulating dangerous objects to avoid preventable accidents and mitigate the potential visual outcomes that may arise as a result.

## Introduction

Ocular trauma is an important preventable cause of monocular vision impairment in children worldwide [1]. When encountering cases of ocular or periocular trauma, it is important to maintain a high index of suspicion for open globe injuries, given the difficulty in diagnosing them due to swelling and potentially hidden wounds. The mechanism of the trauma should always be investigated to understand the extent of the injury. Sharp needle-induced open-globe injuries can sometimes be localized and self-sealing [2]. Interestingly, the entry site of the wound may not be visible during a routine examination. In such cases, imaging modalities like B-scan ultrasonography and MRI can be employed to assess the integrity of the globe and guide the management approach. Exploration under general anesthesia in an operating room is necessary to determine whether the wound is self-sealing. However, it is important to prioritize the immediate management of potentially life-threatening complications, such as orbital cellulitis, before proceeding with surgical exploration [2]. Penetrating needle eye injuries are not documented enough in the literature. There are few reports describing the presentation of such cases [3-5]. This report aims to illustrate the clinical presentation, management, and outcome of a veterinary needle-induced eye injury. The scarcity of literature on ocular trauma caused by veterinary needles serves to highlight the exceptional nature of this case.

## Case presentation

**Figure 1:**
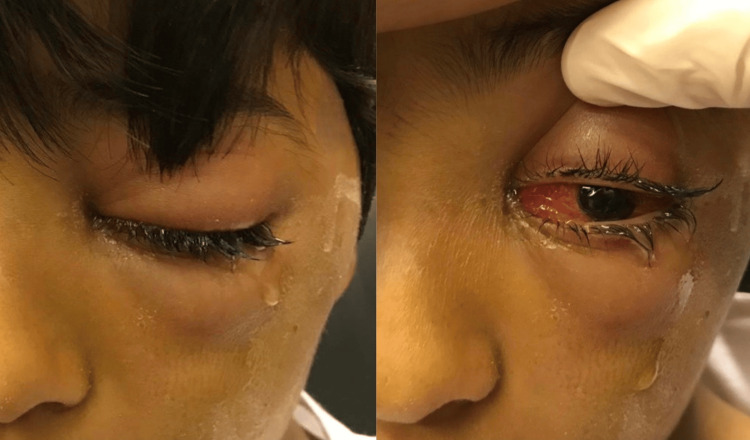
External photo showing left side periorbital and orbital cellulitis at the time of presentation.

A nine-year-old male presented with severe left eye pain, swollen eyelids, and a drop in vision to our ophthalmic emergency room a couple of days after sustaining trauma to the left eye by a soil-contaminated veterinary needle that had been used to inject a sheep. He initially went to an eye clinic a few hours after the trauma and his mother was reassured. On day 3, he presented to an eye center with eyelid swelling. A dilated fundus exam revealed vitreous hemorrhage and retinal detachment with a large retinal tear inferiorly. He was given intramuscular injections of cefuroxime, clindamycin, and metronidazole and was referred to our facility for retinal evaluation. On day 4, the patient arrived at our ophthalmic emergency room, with a history of severe pain and tearing in his left eye, as well as fever and a reduced level of consciousness. A visual acuity test revealed that he had 20/20 vision in his right eye and could only see hand motions in his left eye. Examination of the left eye showed moderate proptosis, periorbital swelling, severe conjunctival chemosis, and severe restriction of ocular movements in all directions especially on adduction with severe pain (Figure 1). 

**Figure 2:**
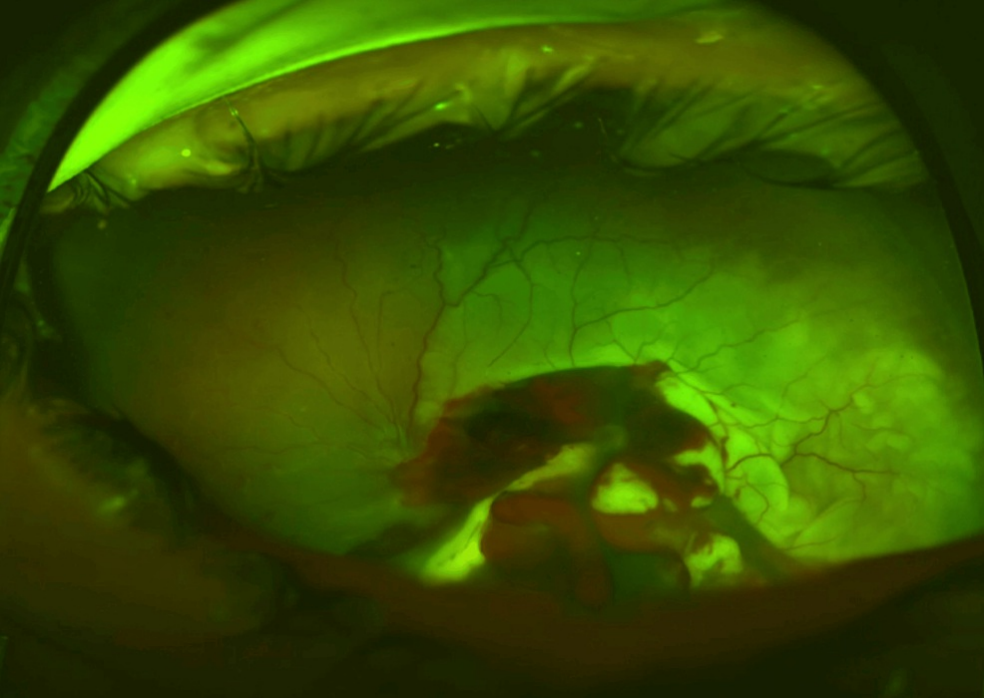
Fundus photo showing retinal detachment and vitreous hemorrhage at presentation.

The slit-lamp examination revealed chemosis of the conjunctiva, but there were no signs of a penetrating injury, and no epithelial injury was observed on fluorescein staining. Additionally, the Seidel test was negative. Intraocular pressures were 15 and 17 mmHg. Examination of the pupils revealed a left dilated regular pupil with a relative afferent pupillary defect (RAPD). The anterior chamber exhibited no signs of inflammation, appearing to be well-formed and deep in nature, with the cornea and lens maintaining a transparent quality. A dilated fundoscopy revealed a rhegmatogenous retinal detachment (RRD) with vitreous hemorrhage (Figure 2).

**Figure 3:**
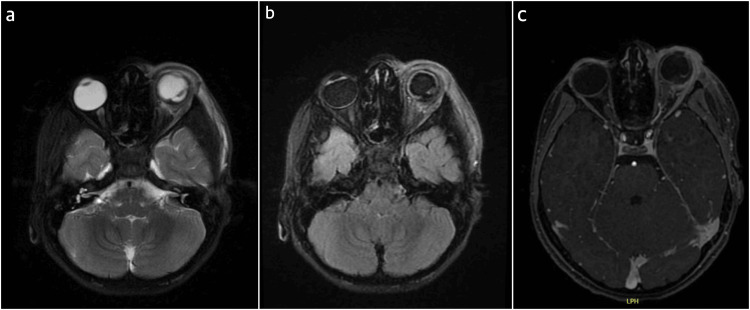
MRI brain and orbits at the time of presentation. Diffuse ocular wall thickening as well as temporal choroidal discontinuity far posteriorly by the equator with subretinal fluid with minimal degree of retraction related to the choroid and retinal area associated with preseptal/postseptal cellulitis with infiltration of the related part of the lacrimal gland, posterior episclera and proximal part of the optic nerve. (a) T2-weighted image. (b) T2 flair with fat suppression. (c) Fat suppressed contrast-enhanced T1-weighted image.

Gentle B-scan showed organized vitreous opacities more along the ocular wall temporally and an ocular wall defect including the retina, choroid, and sclera detected temporal around the equator but no sign of endophthalmitis (heterogenous debris in the vitreous cavity, membranes formation, choroidal thickening, edema of the optic nerve head and thickening of sclera). The results of the blood culture were negative for growth and the white blood cell count demonstrated hyperleukocytosis with a predominance of neutrophils. The conjunctival swab test was not performed in this case. MRI of the brain and orbits was performed and revealed temporal choroidal discontinuity far posteriorly by the equator as well as preseptal/postseptal cellulitis with infiltration of the proximal part of the optic nerve (Figures 3a-3c). The final diagnosis indicated a penetrating globe injury accompanied by traumatic orbital cellulitis and optic nerve infiltration, as well as traumatic retinal detachment.

**Figure 4:**
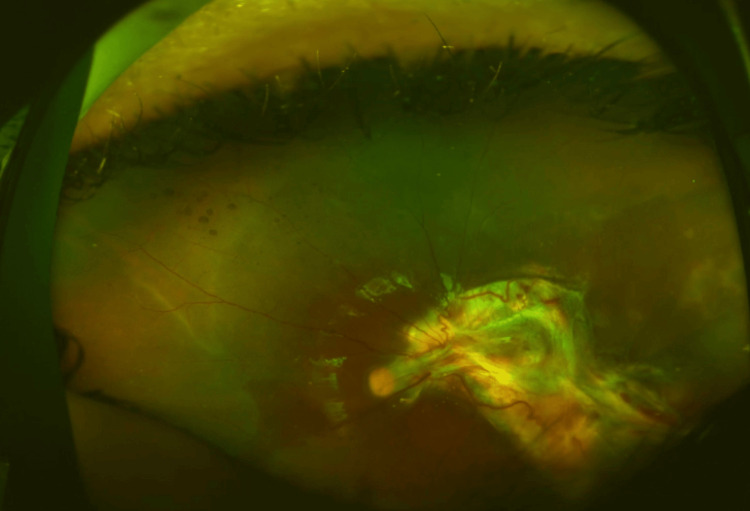
Flat retina under silicon oil two months after surgical treatment with a traction band extending into the entry wound.

The patient was admitted and started on systemic broad-spectrum intravenous antibiotics including vancomycin (40 mg/kg/day), ceftazidime (30 mg/kg/day), and metronidazole (30 mg/kg/day), topical steroid drops 1% four times daily and fourth-generation fluoroquinolone drops four times daily. Systemic steroid (1 mg/kg/day) was initiated five days later, after which the swelling rapidly subsided. Ten days after his presentation, the patient underwent globe exploration and vitreoretinal surgery. The exploration was conducted at the possible site of needle entry in the supratemporal region and revealed conjunctival adhesion over a self-sealed scleral wound with uveal tissue present at 2 o'clock. A vitreoretinal surgeon performed a pars plana vitrectomy to repair the retinal detachment. During the procedure, the posterior hyaloid was found to be truncated and heavily adherent to the macula, with an extension to the inferotemporal area that formed a tractional band. Given the potential risks of opening an apparent exit wound, the traction band was intentionally maintained in its current position. Silicone oil was used to achieve retinal tamponade at the end of the procedure. On subsequent visits, the patient was asymptomatic with complete resolution of orbital cellulitis. His visual acuity reached 20/200 with correction (+9.00/-1.50 x 95). The retina was flat under silicone oil with a thick vascularized membrane over the macular area (Figure 4). He was last seen one year after surgery with stable vision. His examination showed a mild posterior subcapsular cataract and a thick membrane extending from the inferotemporal retina to the macula under silicone oil. He was advised to use protective eyewear for the uninvolved eye.

## Discussion

Ocular trauma can have devastating outcomes. Lack of child supervision by parents when playing outdoors increases the risk of injury. It is important to keep in mind the etiology and mechanism of trauma and keep a high index of suspicion of the open globe even if not suggestive from the history. Making the diagnosis of an open globe can be difficult in cases of periocular swelling especially when the wound is located posteriorly or is occult. It is imperative to obtain a CT scan or B scan ultrasonography if the history of trauma is highly indicative of a possible penetrating injury, given the potential severity of such injuries. Imaging can give information concerning the integrity of the globe, the presence of an intraocular foreign body (IOFB), and the status of the orbital and facial structures [2]. CT scan is the test of choice in cases of trauma, but since our CT scan machine was out of order and an IOFB was ruled out by B-scan, MRI was performed instead. Types of needles reported causing penetrating globe injury include dental needles [5], hypodermic needles [6], sewing needles [4], and needles used in peribulbar and retrobulbar injections [7]. Orbital cellulitis may result from any injury perforating the orbital septum. Orbital inflammation may be noted within 48-72 hours after injury, or, in the case of a retained orbital foreign body, it may be delayed for several months [8]. Orbital cellulitis can result in orbital and intracranial complications such as optic neuropathy, cranial nerve palsies, subperiosteal abscess, orbital abscess, cavernous sinus thrombosis, meningitis, and subdural or brain abscess [9]. There have been reports of orbital cellulitis following peribulbar anesthesia [10,11].

Immediate repair of open globe injury is mandatory, but in our case, the presence of a life-threatening complication (orbital cellulitis and optic nerve infiltration) rendered globe exploration risky. Furthermore, the wound was caused by a needle which was presumed to be self-sealed, and this was supported by imaging.

To our knowledge, no prior studies have described an ocular penetrating trauma by a veterinary needle. We believe the needle perforated the orbital septum temporally and then penetrated the globe, which resulted in orbital cellulitis, open globe, and retinal detachment. Retinal detachment in open globe injuries can be caused by either direct trauma or traction of proliferative vitreous. It is usually associated with poor prognosis. It is a significant predictor of worse final visual outcomes [12]. Despite the needle being contaminated and the injury occurring in a rural location, our patient did not develop endophthalmitis, which is a commonly reported complication of open globe injuries with needles and is considered a significant poor prognostic factor [1,3]. Awareness campaigns aimed at educating families about the risks associated with children handling dangerous objects are essential to prevent such injuries, which can have serious consequences on vision.

## Conclusions

Needles are generally fine and flexible, and a penetrating injury is more likely to self-seal. Prompt decision-making is important for the fate of the traumatized eye. It is widely acknowledged that the immediate repair of open globe injuries is of utmost importance, yet due to our assumption that the wound had self-sealed, exploration of the globe was deferred until the management of vision/life-threatening conditions such as orbital cellulitis had been achieved. This case report highlights the importance of a judicious approach to complex ocular trauma cases. Due to the delay in presentation and management and the presence of complications resulting from the trauma, the prognosis for our patient has been significantly compromised. Despite undergoing treatment, the visual outcome for the patient remained unfavorable, highlighting the need for preventive measures to minimize the occurrence of similar incidents. It is essential to prioritize education and awareness campaigns for families to prevent accidents involving dangerous objects and their potential detrimental effects on vision. We also strongly recommend the following: (1) a high level of suspicion of such cases regardless of the presentation and (2) prompt diagnosis and management or timely and appropriate referral for early intervention to avoid complications.
